# Characteristics associated with occurrence of stroke in patients with infective endocarditis – a retrospective cohort study

**DOI:** 10.1186/s42466-024-00317-4

**Published:** 2024-04-11

**Authors:** H. Schuermann, R. von Rennenberg, C. Riegler, I. Rangus, S. Litmeier, J. F. Scheitz, W. Doehner, H. Audebert, T. B. Braemswig, C. H. Nolte

**Affiliations:** 1 Klinik und Hochschulambulanz für Neurologie, Charité – Universitätsmedizin Berlin, corporate member of Freie Universität Berlin and Humboldt-Universität zu Berlin, Hindenburgdamm 30, 12203 Berlin, Germany; 2 Paracelsus Medical University Salzburg, Salzburg, Austria; 3 Center for Stroke Research Berlin, Charité-Universitätsmedizin Berlin, Berlin, Germany; 4 Berlin Institute of Health (BIH) at Charité - Universitätsmedizin Berlin, Berlin, Germany; 5 DZHK German Centre for Cardiovascular Research (DZHK), Partner-Site Berlin, Berlin, Germany; 6 German Heart Center of the Charite, Campus Virchow, Charité-Universitätsmedizin Berlin, Berlin, Germany; 7 Berlin Institute of Health-Center or Regenerative Therapies, Universitätsmedizin Berlin, Berlin, Germany

**Keywords:** Infective endocarditis, Acute stroke, Risk factors, Prognostic factors, Systemic complications, Congenital heart disease, Dental focus

## Abstract

**Background:**

Stroke is a severe complication of infective endocarditis (IE), associated with high rates of mortality. Data on how IE patients with and without stroke differ may help to improve understanding contributing mechanisms.

**Methods:**

All patients treated for IE between 2019 and 2021 with and without associated stroke were identified from the medical records of three academic tertiary care hospitals in Germany, all part of Charité – Universitätsmedizin Berlin, Germany. Multivariable logistic regression analyses were performed to identify variables associated with the occurrence of stroke.

**Results:**

The study population consisted of 353 patients diagnosed with IE. Concomitant stroke occurred in 96/353 (27.2%) patients. Acute stroke was independently associated with co-occurring extracerebral arterial embolism [adjusted Odds ratio (aOR = 2.52; 95% confidence interval (CI) 1.35–4.71)], acute liver failure (aOR = 2.62; 95% CI 1.06–6.50), dental focus of infection (aOR = 3.14; 95% CI 1.21–8.12) and left-sided IE (aOR = 28.26; 95% CI 3.59-222.19). Stroke was found less often in IE patients with congenital heart disease (aOR = 0.20; 95% CI 0.04–0.99) and atypical pathogens isolated from blood culture (aOR = 0.31; 95% CI 0.14–0.72).

**Conclusions:**

Stroke is more likely to occur in individuals with systemic complications affecting other organs, too. Special attention should be addressed to dental status. The low incidence of stroke in patients with congenital heart disease may reflect awareness and prophylactic measures.

## Background

Stroke is a potentially devastating complication of infective endocarditis (IE) and associated with worse outcome, especially death [1, 2]. The significance of factors contributing to the complication of stroke in IE is not yet entirely understood. Thus, there is still room for improvement regarding management of the disease and preventive measures of stroke in IE [3]. Data on how patients with IE who have a stroke differ from IE patients without a stroke are scarce. However, such data may help to better understand underlying mechanisms that lead to stroke in IE and support clinical decision making. Previous reports have almost exclusively focused on the pathogens and valves involved, as well as the size of valvular vegetations [4–8]. Data on predisposing factors, accompanying diseases and primary site of infection in IE patients with stroke are limited, but may help to provide more individualized care of IE patients.

This study therefore aimed to identify factors that are independently associated with occurrence of stroke in patients diagnosed with IE.

## Methods

In this observational study, adult patients diagnosed with IE treated at three tertiary care academic hospitals, all part of Charité – Universitätsmedizin Berlin, Germany between 2019 and 2021 were analyzed. Acute stroke (both ischemic and hemorrhagic) during the hospital stay was defined as the primary outcome. We compared IE patients with any stroke (ischemic and/or hemorrhagic) because both entities commonly occur simultaneously in IE patients. In order to avoid statistical type 2 error due to small sample sizes, we did not further differentiate between ischemic and hemorrhagic stroke.

### Patients

Patients were retrospectively identified via the hospital record system using the ICD-10 code for IE (ICD-10 I33.0). Inclusion criteria were age ≥ 18 years, inpatient treatment, IE as main diagnosis according to the treating physicians’ discharge letter. We reviewed all cases regarding the modified Duke’s criteria for IE and excluded patients who did not fulfill the Duke’s criteria from our analyses.^9^ Exclusion criteria were local infection of intracardiac devices or implanted material without involvement of the cardiac valves and sole outpatient-appointments.

### Clinical data

Clinical data was collected from the routine medical records. Data on baseline characteristics and comorbidities such as cardiovascular risk factors, congenital heart disease, prosthetic heart valves, intracardiac medical devices (other than artificial heart valves, e.g. MitraClip, ICD, pacemaker) and preexisting heart disease were extracted. In addition, data on the focus of infection, causative pathogen, affected heart valves and other organ manifestations were collected. We defined different groups of pathogens isolated from blood cultures based on the major Duke criterion [9]: Staphylococcus aureus, Staphylococcus epidermidis, Streptococcus mitis/oralis, Enterococcus faecalis and atypical pathogens. There was only one case of IE caused by a HACEK bacterium. We therefore did not include HACEK pathogens as a separate category in our analyses. The clinical diagnosis of stroke (ischemic, hemorrhagic or both) was based on imaging findings (MRI, CT) and clinical neurological assessments. Diagnosis of ischemic stroke was established if acute diffusion restriction was visible on MRI or infarct typical hypo-attenuation was seen on CT and described in radiological findings and/or medical records. Diagnosis of hemorrhagic stroke was established if a susceptibility imaging sequence (either T2* or susceptibility weighted imaging (SWI)) showed an intracranial hemorrhage on MRI or CT showed hyperintense lesion compatible with intracranial hemorrhage. With respect to lesion location in the brain, three different vascular supply areas were differentiated on MRI / CT: 1. the territory of the left internal carotid artery, 2. the territory of the right internal carotid artery, and 3. the vertebrobasilar territory [10]. Information on vascular status of stroke patients was drawn from the radiologic evaluation of angiography (CTA/MR) and/or duplex ultrasound.

### Statistics

The chi-squared test and Fisher’s exact test were used for comparisons of nominal variables. The Mann–Whitney U test was used for comparisons of continuous variables. Variables suggesting an association with *p* < 0.1 in univariable analysis were selected for multivariable logistic regression analysis for the primary outcome (i.e. acute stroke). Statistical significance was determined at an α level of 0.05. Statistical analyses were performed using SPSS version 28.0 (SPSS Inc., Chicago, IL).

## Results

**Fig. 1:**
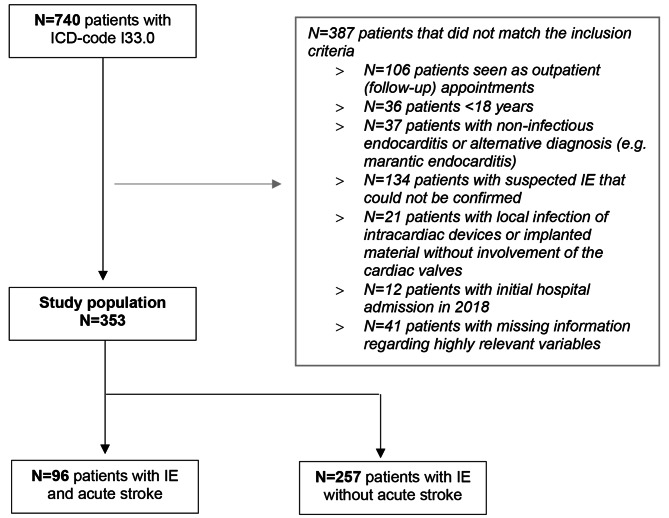
Flowchart of patient selection. Notes: IE: Infective endocarditis

Using the ICD-10 code I33.0 (IE), we identified 740 patients diagnosed with IE of whom 353 fulfilled the inclusion criteria and therefore qualified for the final analysis (see Flowchart, Fig. 1). Of these, 71.7% were male and the median age was 69 years (IQR, 56–77)]. IE affected mainly the valves on the left side of the heart: the aortic valve was affected most frequently (217/353; 61.5%), followed by the mitral valve (118/353; 33.4%), the tricuspid valve (32/353; 9.1%) and the pulmonary valve (18/353; 5.1%). In 37/353 (10.5%) patients, more than one heart valve was affected. Brain imaging was performed in the majority of IE patients (230/353; 65.2%). Acute ischemic and/or hemorrhagic brain lesions were detected in 96/230 of patients with brain imaging (41.7%). Most patients with acute brain lesions on imaging had focal neurologic deficits (76/96; 79.2%). Of patients without focal neurologic deficits and brain imaging (N = 20), six (30%) had proof of stroke. Signs of systemic sequelae were common with 22.1% of IE patients suffering concomitant extracerebral arterial embolisms, 21.5% having acute kidney failure requiring hemodialysis and 10.8% having acute liver failure. During hospitalization 75/353 (21.2%) patients died. Details are shown in Table 1.

**Table 1:** Baseline characteristics of IE patients with stroke and without stroke (univariable analysis)

Variable	All patients^a^ (*N* = 353)	No stroke^a^ (*N* = 257)	IE and stroke^a^ (*N* = 96)	*p*-value^b^	Odds Ratio^c^	CI (95%)^c^
Age, years, (median, IQR)	69 [56–77]	69 [56; 77]	68 [59; 78]	0.989	1.00	0.99–1.02
Sex, male (*n*, %)	253 (71.7%)	179 (69.6%)	74 (77.1%)	0.168	1.47	0.85–2.53
Vascular risk factors						
Arterial hypertension	258 (73.1%)	188 (73.2%)	70 (72.9%)	0.965	0.99	0.58–1.68
Dyslipidemia	157 (44.5%)	112 (43.6%)	45 (46.9%)	0.579	1.14	0.71–1.83
Diabetes mellitus	99 (28.0%)	69 (26.8%)	30 (31.3%)	0.413	1.24	0.74–2.07
Coronary artery disease	115 (32.6%)	86 (33.5%)	29 (30.2%)	0.562	0.86	0.52–1.43
Atrial fibrillation	110 (31.2%)	84 (32.7%)	26 (27.1%)	0.312	0.77	0.46–1.29
Preexisting antithrombotic treatment	212 (60.1%)	159 (61.9%)	53 (55.2%)	0.256	0.76	0.47–1.22
Previous stroke	54 (15.3%)	40 (15.6%)	14 (14.6%)	0.820	0.93	0.48–1.79
Congenital heart disease (e.g. bicuspid aortic valve)	28 (7.9%)	26 (10.1%)	2 (2.1%)	0.013	**0.19**	**0.04–0.81**
Prosthetic heart valve	135 (38.2%)	102 (39.7%)	33 (34.4%)	0.361	0.80	0.49–1.30
Intracardiac medical devices other than artificial heart valves (e.g. MitraClip, ICD, pacemaker)	74 (21.0%)	63 (24.5%)	11 (11.5%)	0.007	**0.40**	**0.20–0.79**
Previous heart disease other than congenital heart disease	111 (31.4%)	88 (34.2%)	23 (24.0%)	0.064	0.61	0.35–1.03
Pathogen isolated in blood culture				0.013	**0.79**	**0.69–0.89**
Staphylococcus aureus	91 (25.8)	56 (21.8%)	35 (36.5%)			
Staphylococcus epidermidis	37 (10.5%)	31 (12.1%)	6 (6.3%)			
Enterococcus faecalis	37 (10.5%)	27 (10.5%)	10 (10.4%)			
Staphylococcus mitis/oralis	24 (6.8%)	19 (7.4%)	5 (5.2%)			
Atypical	118 (33.4%)	97 (37.7%)	21 (21.9%)			
No pathogen found	46 (13.0%)	27 (10.5%)	19 (19.8%)			
Affected side of heart						
Left heart affected	307 (87.0%)	212 (82.5%)	95 (99.0%)	< 0.001	**20.17**	**2.74-148.46**
Right heart affected	50 (14.2%)	49 (19.1%)	1 (1.0%)	< 0.001	0.05	0.01–0.33
Focus of infection identified	127 (36.0%)	94 (36.6%)	33 (34.4%)	0.618	0.88	0.54–1.45
Dental focus of infection	29 *(8.2%)*	17 (6.6%)	12 (12.5%)	0.073	**2.59**	**1.07–6.26**
Spondylodiscitis as focus of infection	25 (*7.1%)*	17 (6.6%)	8 (8.3%)	0.575	1.28	0.54–3.08
Catheter associated focus of infection	18 *(5.1%)*	15 (5.8%)	3 (3.1%)	0.303	0.52	0.15–1.84
Other organ manifestations						
Spondylodiscitis	29 (8.2%)	21 (8.2%)	8 (8.3%)	0.961	1.02	0.44–2.39
Metastatic abscess	41 (11.6%)	28 (10.9%)	13 (13.5%)	0.490	1.28	0.63–2.59
Extracerebral arterial embolic events	78 (22.1%)	42 (16.3%)	36 (37.5%)	< 0.001	**3.07**	**1.81–5.21**
Acute kidney failure requiring HD	76 (21.5%)	49 (19.1%)	27 (28.1%)	0.065*	1.66	0.97–2.86
Acute liver failure	38 (10.8%)	19 (7.4%)	19 (19.8%)	< 0.001	**3.09**	**1.56–6.14**

Notes: IE indicates infective endocarditis; ICD, implantable cardioverter defibrillator; IVDA, intravenous drug abuse; HD, hemodialysis. ^a^Absolute frequencies (relative frequencies in %), median [25 and 75 percentiles]. ^b^Pearson χ^2^ test or Fisher’s exact test for nominal variables, Mann-Whitney-U test for categorical and continuous variables. ^c^Unadjusted odds ratios and 95% confidence intervals were calculated using Pearson χ^2^ or Fisher’s exact test for nominal variables and unadjusted logistic regression analysis for continuous variables

### Comparison of IE patients with and without stroke

Acute stroke was diagnosed in 96/353 (27.2%) patients [77.1% male, median age 68 (IQR, 59–78) years]. Of patients with an acute stroke, the majority had an ischemic stroke (63/96; 65.6%), or both an ischemic and a hemorrhagic stroke (25/96; 26.0%). Secondary hemorrhagic transformation was seen in 17 patients with ischemic stroke (19.3%). Isolated hemorrhagic stroke occurred in only a few patients (8/96; 8.3%). In most IE patients with stroke, more than one vascular supply area was affected (65/96; 67.7%). All three vascular supply areas were affected in 43/96 patients (44.8%). Stroke was detected with similar frequencies in the three vascular supply areas (territory of the left internal carotid artery 70.8%, territory of the right internal carotid artery 74.0%, vertebrobasilar territory 67.7%).

Considering concurrent stroke etiologies, there was no significant difference regarding vascular risk factors (atrial fibrillation, dyslipidemia, diabetes mellitus, previous stroke) between IE patients with and without stroke (see Table 1). Extracranial atherosclerotic plaques with relevant stenosis (> 50%) could be detected in eight IE patients with stroke. Extracranial stenosis was symptomatic (ipsilateral to the vascular territory) in two patients with stroke. The remaining six patients had either proof of stroke in all three major vascular territories or stenosis was not ipsilateral (asymptomatic stenosis). Intracranial atherosclerotic plaques were seen in nine patients, but only three showed relevant intracranial stenosis (> 50%). No patient had lacunar stroke.

In the univariable analysis, IE patients with an acute stroke more often had IE on the left side of the heart (i.e. aortic and/or mitral valve) (99.0% vs. 82.5%; *p* < 0.001), acute liver failure (19.8% vs. 7.4%; *p* < 0.001), and concomitant extracerebral arterial embolism (37.5% vs. 16.3%; *p* < 0.001) compared to IE patients without stroke. Acute kidney failure requiring hemodialysis was numerically more common in IE patients with stroke (28.1% vs. 19.1%; *p* = 0.065). Congenital heart disease (2.1% vs. 10.1%; *p* = 0.013), and intracardiac medical devices other than artificial heart valves [e.g. MitraClip, ICD, pacemaker (11.5% vs. 24.5%; *p* = 0.007)] were less prevalent in patients with stroke. The numerical difference in prevalence of previous heart disease other than congenital heart disease (24.0% vs. 34.2%; *p* = 0.064) just failed to reach statistical significance. Dental focus of infection was numerically twice as common in IE patients with stroke (12.5% vs. 6.6%; *p* = 0.073). Of note, no focus of infection could be identified in the majority of our study population (see Table 1). The spectrum of pathogens isolated in blood culture differed significantly between IE patients with and without stroke (*p* = 0.013). Details are listed in Table 1.

In the multivariable analysis, factors independently associated with acute stroke were concomitant extracerebral arterial embolism (aOR = 2.52; 95% CI 1.35–4.71), acute liver failure (aOR = 2.62; 95% CI 1.06–6.50), dental focus of infection (aOR = 3.14; 95% CI 1.21–8.12) and IE on the left side of the heart (aOR = 28.26; 95% CI 3.59-222.19). On the contrary, congenital heart disease was independently associated with a lower risk of stroke (aOR = 0.20; 95% CI 0.04–0.99), in addition, atypical pathogens isolated from blood culture were independently associated with lower risk of stroke as well (aOR = 0.31; 95% CI 0.14–0.72). Refer to Table 2 for details.

**Table 2:** Multivariable analysis of factors associated with stroke

Variable	Results^a^ (no stroke) (*N* = 257)	Results^a^ (stroke) (*N* = 96)	*p*-value	adjusted Odds Ratio	CI (95%)
Left heart affected (mitral and/or aortic valve)	212 (82.5%)	95 (99.0%)	0.001	**28.26**	**3.59-222.19**
Extracerebral arterial embolic events	42 (16.3%)	36 (37.5%)	0.004	**2.52**	**1.33–4.71**
Acute liver failure	19 (7.4%)	19 (19.8%)	0.037	**2.62**	**1.06–6.50**
Dental focus of infection	17 (6.6%)	12 (12.5%)	0.019	**3.12**	**1.21–8.12**
Intracardiac medical devices other than artificial heart valves (e.g. MitraClip, ICD, pacemaker)	63 (24.5%)	11 (11.5%)	0.055	0.46	0.21–1.02
Congenital heart disease (e.g. bicuspid aortic valve)	26 (10.1%)	2 (2.1%)	0.049	**0.20**	**0.04–0.99**
Previous heart disease other than congenital heart disease	88 (34.2%)	23 (24.0%)	0.139	0.62	0.33–1.17
Acute renal failure requiring HD	49 (19.1%)	27 (28.1%)	0.949	0.98	0.49–1.96
Pathogen isolated in blood culture (no pathogen found = ref)					
Staphylococcus aureus	56 (21.8%)	35 (36.5%)	0.876	0.94	0.41–2.15
Staphylococcus epidermidis	31 (12.1%)	6 (6.3%)	0.051	0.32	0.10-1.00
Streptococcus mitis/oralis	19 (7.4%)	5 (5.2%)	0.137	0.36	0.10–1.38
Enterococcus faecalis	27 (10.5%)	10 (10.4%)	0.610	0.77	0.28–2.14
Other	97 (37.7%)	21 (21.9%)	0.006	**0.31**	**0.14–0.72**

Notes: IE indicates infective endocarditis; ICD, implantable cardioverter-defibrillator; HD, hemodialysis. ^a^Absolute frequencies (relative frequencies in %)

We conducted a sensitivity analysis specifically focusing on the subgroup of patients with IE affecting the left side of the heart alone, given that this variable exhibited the strongest association with the occurrence of stroke. The sensitivity analysis showed that associations found in the whole group of IE patients remained significant for all variables besides acute liver failure. For details, see Table 3.

**Table 3:** Sensitivity analysis of patients with IE affecting the left side of the heart

Variable	Results^a^ (no stroke) (*N* = 212)	Results^a^ (stroke) (*N* = 95)	*p*-value	Odds Ratio	CI (95%)
Extracerebral arterial embolic events	34 (16.0%)	35 (36.8%)	0.005	**2.45**	**1.31–4.60**
Acute liver failure	15 (7.1%)	18 (18.9%)	0.065	2.37	0.95–5.91
Dental focus of infection			0.016	**3.30**	**1.25–8.72**
Intracardiac medical devices other than artificial heart valves (e.g. MitraClip, ICD, pacemaker)	53 (25.0%)	10 (10.5%)	0.030	**0.41**	**0.18–0.92**
Congenital heart disease (e.g. bicuspid aortic valve)	15 (7.1%)	2 (2.1%)	0.050	0.20	0.04–0.99
Previous heart disease other than congenital heart disease	75 (35.4%)	23 (24.2%)	0.183	0.65	0.35–1.23
Acute renal failure requiring HD	39 (18.4%)	26 (27.4%)	0.891	0.95	0.47–1.92
Pathogen isolated in blood culture (no pathogen found = ref)					
Staphylococcus aureus	39 (18.4)	34 (36.2%)	0.850	0.92	0.40–2.13
Staphylococcus epidermidis	28 (13.2%)	6 (6.4%)	0.057	0.32	0.10–1.03
Streptococcus mitis/oralis	13 (6.1%)	5 (5.3%)	0.128	0.35	0.09–1.35
Enterococcus faecalis	24 (11.3%)	10 (10.6%)	0.609	0.77	0.27–2.14
Other	85 (40.1%)	20 (21.3%)	0.006	**0.31**	**0.13–0.71**

Notes: IE indicates infective endocarditis; ICD, implantable cardioverter-defibrillator; HD, hemodialysis. ^a^Absolute frequencies (relative frequencies in %)

## Discussion

In our large retrospective cohort comprising 353 IE patients from three different tertiary care hospitals, about 3 in 10 individuals had an acute stroke. Multivariable analysis identified several factors that were associated with the occurrence of acute stroke in IE patients. First, stroke occurred more commonly in patients with acute liver failure and concomitant extracerebral arterial embolisms, reflecting multi-organ manifestations. Second, dental focus was associated with stroke. Third, preexisting congenital heart disease was less frequently associated with stroke as complication of IE. Fourth, atypical pathogens were more commonly found in IE patients that did not suffer acute stroke. Fifth, stroke occurred more commonly in patients with IE on the left side of the heart. Our findings on multi-organ manifestations, dental focus and preexisting risk constellations were robust in sensitivity analysis excluding patients with IE affecting the right side of the heart only. The effect sizes indicated a marked association with all aORs either above 2 (in case of a positive association) or below 0.5 (in case of a negative association).

With respect to multi-organ manifestations (i.e. accompanying liver failure, concomitant arterial emboli), the occurrence of embolic complications in the brain may reflect an elevated overall likelihood of embolic complications throughout the entire organism. Liver failure may either reflect a decompensating septic situation inducing immunothrombosis or additional coagulation disorders leading to thrombotic emboli or both.^11^ Our data corroborate that IE is a cardiac disease with substantial systemic complications.^12–15^

Interestingly, a dental infection focus in IE was also associated with a higher likelihood of stroke. A dental focus may go along with more systemic complications during the course of IE, because involved pathogens may be more difficult to eradicate or incorporate more virulent properties.^16,17^ Oral microorganisms (commensal as well as pathogenic) can easily gain access to the systemic bloodstream when there is a breach in the oral mucosal barrier, causing bacteremia. Due to adhesive properties of some of these microorganisms and their capacity to build biofilms, they play an important role in the formation of vegetations, the bacterial load, and thus systemic complications.^18,19^

Of note, congenital heart disease was associated with less frequent occurrence of stroke in IE. The protective association may be explained by an increased awareness for IE.^9^ This may prompt early and more aggressive treatment of IE, which in turn could prevent a more fulminant course of the disease and consequently reduce likelihood of occurrence of complications like embolic events. ^9,20,21^

Additionally, atypical pathogens of IE were found less frequently in blood cultures of stroke patients. Atypical pathogens may represent a group of less aggressive microorganisms that do not cause such a severe course of disease as for example Staphylococcus aureus. Independent association with a category of pathogens isolated in blood culture may indicate different pathogen spectrum in IE patients with vs. without stroke.

The high proportion of patients with IE affecting the valves on the left side of the heart is in line with typical other IE cohorts.^14,22,23^ Our results confirm that stroke associated with IE affecting the left side of the heart is common due to anatomical reasons.

While our analysis presents a relatively large study population with detailed work-up, limitations have to be taken into account. Inherent to the retrospective study design and university hospital setting, selection bias, bias by indication (to perform diagnostic tests) and the time period of interest may limit our results. In particular, bias by indication to perform brain imaging applies. Since brain imaging (CT/MRI) was not mandatory in all patients during IE work-up, but rather particularly in patients with neurologic symptoms, silent strokes (stroke not causing overt clinical symptoms) may have been missed in some patients. In addition, IE patients without severe complications may be diagnosed less commonly and may therefore be underrepresented in our study cohort. Moreover, we did not distinguish patients with ischemic from those with hemorrhagic stroke. Characteristics may differ in these patients. However, nearly one in three stroke patients had imaging proof of both ischemic and hemorrhagic stroke at the same time, introducing a third category. Larger study samples are necessary to produce sample sizes that would allow to analyze the entities separately. The assessed time period includes the COVID-19 pandemic which might have influenced patients’ behavior regarding their health care behavior (e.g. preventive check-ups, time until presentation to a doctor) and hospital work flows.^24,25^

## Conclusions

In conclusion, stroke in IE occurs more frequently in patients with systemic complications involving multiple organs. IE should be recognized as a cardiac disease with systemic complications, commonly affecting the liver, kidneys and brain, too. Our data stress the importance of the oral cavity as a potential source of bacteremia. Hence, identification and treatment of a dental focus should be an essential part of medical work-up in every IE. Finally, the lower rate of stroke in IE patients with congenital heart disease may reflect higher awareness resulting in better prophylactic measures and earlier treatment in these patients.

## Data Availability

The datasets generated, used and analyzed during the current study are available from the corresponding author on reasonable request.

## Abbreviations

IE: Infective endocarditis
aOR: Adjusted Odds Ratio
CI: Confidence Interval
CT: Computed Tomography
MRI: Magnetic Resonance Imaging
ICD: Implantable cardioverter–defibrillator
IQR: Interquartile range
